# Consistent condom utilization and associated factors among HIV positive clients attending ART clinic at Pawi general hospital, North West Ethiopia

**DOI:** 10.1371/journal.pone.0261581

**Published:** 2021-12-21

**Authors:** Alex Yeshaneh, Adugna Lencha, Amlaku Mulat Aweke, Yaregal Dessalew, Tegegne Wale, Esubalew Mekuriya, Temkin Abdulahi, Alemu Workineh, Meseret Yitayew, Hirut Dinku, Genet Asfaw

**Affiliations:** 1 Department of Midwifery, College of Medicine and Health Science, Wolkite University, Wolkite, Ethiopia; 2 Department of Midwifery, Institute of health science, Pawi Health Science College, Pawi, Ethiopia; 3 Department of Midwifery, College of Medicine and Health Science, Bahirdar University, Bahirdar, Ethiopia; 4 Department of Midwifery, College of Medicine and Health Science, Assosa University, Assosa, Ethiopia; 5 Department of Midwifery, College of Medicine and Health Science, Debretabor University, Debretabor, Ethiopia; 6 Department of Reproductive Health, College of Medicine and Health Science, Hawassa University, Hawassa, Ethiopia; 7 Department of Nursing, College of Medicine and Health Science, Assosa University, Assosa, Ethiopia; University of Tennessee Health Science Center College of Pharmacy Memphis, UNITED STATES

## Abstract

**Background:**

Human immunodeficiency virus (HIV) affects a highly significant number of people and is responsible for the deaths of many people in sub-Saharan African countries alone. The best prevention method for this virus is through consistent condom utilization which can help to prevent drug-resistant HIV infection and acquisition of new infection. Therefore, this study aimed to assess consistent condom utilization and associated factors among HIV-positive individuals attending an antiretroviral therapy clinic at Pawi general hospital, North West Ethiopia in 2020.

**Methods:**

An institutional based cross-sectional study was conducted among 419 HIV-positive individuals who have follow-up in the Pawi general hospital antiretroviral therapy clinics, from January to February 2020. The study subjects were reached using a systematic sampling technique and data were collected using a pretested and structured questionnaire. Data entry and analysis were performed using epi-data version 3.1 and SPSS version 23 respectively. Binary and multivariable analyses with a 95% confidence level were performed. In the final model, variables with P < 0.05 were considered statistically significant.

**Results:**

A total of 419 antiretroviral therapy study participants were participated in the study with a response rate of 100%. In this finding, the consistent condom utilization rate was 49.2% [95% CI: 42.2–56.5%]. After controlling for possible confounding factors, the results showed that place of residence [AOR = 2.16, 95% CI: 1.05, 4.45], marital status [AOR = 0.19, 95%CI: 0.05, 0.67], number of partners [AOR = 0.19, 95% CI: 0.07, 0.55] and level of education [AOR = 5.33, 95% CI: 1.57, 18.08] were associated factors of consistent condom utilization.

**Conclusion:**

Consistent condom utilization among HIV-positive clients attending antiretroviral therapy clinics at Pawi general hospital was low. Residence, marital status, level of education and number of partners were significantly associated factors of consistent condom use. Health education program and counseling services should be started to increase knowledge about way of transmission and appropriate use of condoms, increase self-efficacy towards condom use and reduction in the number of sexual partners.

## Introduction

In 2020 WHO reported, Human Immunodeficiency Virus (HIV)/ Acquired Immune Deficiency Syndrome (AIDS) infected an estimated 37.7 million people worldwide and 25.4 million from Africa alone. In 2021, 680 000 people died from HIV-related causes and 1.5 million people acquired HIV [1]. In Ethiopia, an estimated 800,000 people live with HIV/AIDS, and the prevalence of HIV/AIDS in the general population is estimated to be 1.5% [2].

Antiretroviral therapy (ART) and consistent use of condom has decreased the mortality and morbidity of HIV disease, improving the health of people living with HIV and enables many HIV-infected people to live a longer and healthier life [3]. Previous studies recommended that since highly active antiretroviral therapy became available, the prevalence of unprotected sex and the incidence of sexually transmitted infections (STIs) including HIV have increased [4].

Inconsistent use of a condom by people living with HIV/AIDS (PLWHA) on ART has led to further risk of the HIV infection epidemic and the development of reinfection with new drug-resistant viral strains [5]. The use of condom protects the transmission of HIV when used correctly and consistently. However, many HIV infected people do not use condom regularly which leads to new HIV infection and re-infection [6].

Inconsistent condom use among person on ART is a major public health concern because of the risk of HIV transmission, disruption of HIV service during covid-19 and slowing the public health response to HIV [1, 7]. One of the key methods of preventing HIV/AIDS transmission is the consistent use of condoms during all types of sexual intercourse. The WHO recommends that a specific strategy to evaluate and limit human immunodeficiency virus drug-resistant (HIVDR) be included in all national HIV prevention and treatment plans [8].

According to the Ethiopian Demographic Health Survey (EDHS) 2016 report, about 2% of PLWH had sexual intercourse in the past 12 months with a person who was neither their husband nor lived with them. Among women with a non-marital, non-cohabiting partner, 20% reported using a condom during the last sexual intercourse [9]. HIV programs are focused on prevention efforts for people who are still uninfected with HIV but since the emergence of COVID-19, these programs have not received much attention. Studies show that HIV transmission among HIV positive people is a major problem [7].

Strategies targeted to reduce the infectiousness of HIV-positive individuals by limited secondary HIV transmission should be part of the prevention policy. Although, free condoms are distributed and behavioral changes made by Ethiopian governmental health institutions throughout the country, the emphasis has been on people uninfected with HIV. Therefore, supporting the prevention of HIV transmission is consistent condom utilization which can help to prevent drug resistant HIV infection in patients [10].

HIV drug-resistant virus is the most important problem in the control and treatment of HIV patients. In recent years there has been an increase in the number of patients who are on the second line drugs and have poor disease progression and even married couples are affected by the exchange of different strains of the virus [11]. To attain the extreme protective effect, condoms must be used always correctly and consistently but in Sub-Saharan Africa use is still low and inconsistent. Complaints to use condoms are extensive due to misconceptions, culture, religion, stigma and other determinants. Furthermore, programs and strategies that address and provide free condoms, do not always give how to use correctly, or about rupture and slippage that can occur [12–14]. There is also a gap in determining the magnitude of condom use among people on ART and some important factors. This Study aimed to describe consistent condom utilization and associated factors among people on ART; which enables the patients for better health outcomes and protect others. The patients will have also the advantage of not only preventing resistance virus but also prevention of other STIs, unwanted and unplanned pregnancy.

## Materials and methods

### Study design and setting

An institution-based quantitative cross-sectional study was conducted from January 15/2020 to February 15/2020. The study was done in Pawi general hospital ART follow-up clinic, the hospital has been providing services since 1985. Pawi town is found in the northwest and located approximately 526 km away from the capital city of Ethiopia, Addis Ababa and 421 Km Benishangul Gumuz regional states, Assosa city. The town is bordered by Jawi from the north, Dangur from the northwest and Gilgel Beles from the northeast. Pawi town has 20 kebeles (a smallest administrative unit in Ethiopia which consist at least 500 families or the equivalent of 3,500 to 4,000 persons) with a total population of 89807 (44960 males and 44847 females). The town has one governmental hospital and two health centers. In this hospital, 836 patients were included in the ART follow-up clinic.

### Source and study population

All people who were HIV-positive and received ART follow-up care at Pawi general hospital. Study populations were systematically selected from people living with HIV/AIDS who attended ART clinic at the time of data collection in Pawi general hospital.

### Eligibility criteria

ART attendees aged 18 years and older and had at least one visit to the hospital of ART unit. Those who are mentally ill and unable to communicate verbally were excluded.

### Sample size determination

The sample size was calculated using a single population proportion sample size calculation formula. There was a previous study done in Kolladiaba health center; a prevalence of 55% was taken to calculate the sample size [15].

The sample size of this study was calculated as follows.

N = (Z α /2) ^2^ * P (1-P)/d^2^ = 381

                Z α /2 = Critical value for normal distribution at 95% confidence interval which equals to 1.96 (Value at alpha = 0.05)

                P = 55% was condom utilization in HIV positive peoples

                D = 0.05 was the margin of error.

Add 10% non-response rate = 38

        Required sample size = 381 + 38 = 419

The final total sample size to be included in the study was 419 subjects

### Sampling procedure

Systematic random sampling was used. First, the average number of clients who visit the ART unit daily during the data collection period were estimated based on the previous daily client flow of the units. This was obtained by referring client’s registration book for a month before data collection. Currently, on average 35–40 clients visit the ARV treatment units daily and 836 PLWHA are enrolled in ART during one month of data collection. The calculated sample size is 419 giving k^th^ values of 2. Every two, clients were interviewed throughout the data collection period.

### Data collection procedure

A structured interview questionnaire was first prepared in English then translated into the national language Amharic. Then, the questionnaire was back-translated to English from Amharic by third person. The questionnaire was administered by trained data collectors who were working in study area. Participants were interviewed in an isolated, private room found close to the ART clinic.

### Data quality management

The questionnaire was pre-tested on 5% patients who are attending ART clinic in Gilgel beles health center. It was done to make sure that the questions were consistent with regard to language clarity, easy understandability, coherence, completeness and organization. After pretest, the questionnaire was amended accordingly. Training for data collectors on the data collection and sampling technique were given before start of actual data collection. Investigators and supervisors were checking and reviewing the completed questionnaires on daily basis to ensure completeness and consistency of the information.

### Data processing and analysis

Data was cleaned, coded and entered in to Epi-data 3.1 [16] and exported to SPSS version 23 [17] for further analysis. Descriptive statistics like frequencies and cross tabulation was performed. Binary logistic regression was employed to identify association, and multivariable logistic regression model was used to control the effect of confounder. Variables with p-value <0.2 in the binary analysis was fitted in to the multivariable logistic regression model. Odds ratios (OR), 95% confidence level (CI) and p-values were calculated. Variable with p-value < 0.05 in the multivariable logistic regression analysis was considered as associated factors for consistent condom utilization among HIV positive individuals.

### Ethical approval and consent to participant

Ethical clearance was obtained from the Institutional Review Board (IRB) of Bahir Dar University College of Medicine and Health sciences. Permission to conduct the study was also obtained from Pawi health office. Participants were informed about the purpose and objective of the study. They were also informed that they have the right to discontinue or refuse to participate in the study if they were not comfortable with the questionnaire. Informed written consent was obtained from each study participant. Confidentiality of information and privacy has been observed.

## Result

### Socio-demographic characteristics of the study participants

A total of 419 study subjects were participated in the study with response rate of 100%. Among study participants, one-third (33.7%) were in the age category of 25–34 years. The mean (±SD) age of the study participants were 26.99 (±9.7) years. About two third (62.8%) of the participants were female. More than half of the respondents (55.6%) were from urban and 80.4% were Orthodox religion followers. About 26.5% of the study participants were housewives, 59.9% were married and 43.2% were able to read and write (Table 1).

**Table 1:** Socio-demographic characteristics of consistent condom use HIV infected persons in condom use, Pawi general hospital, North West Ethiopia 2020 (n = 419).

Characteristics	Frequency	Percent
**Age**		
18–24	49	11.7
25–34	134	32
35–44	130	31
≥45	113	25.3
**Sex**		
Female	263	62.8
Male	156	37.2
**Religion**		
Orthodox	337	80.4
Muslim	49	11.7
Protestant	22	5.3
Catholic	11	2.6
**Residence**		
Urban	233	55.6
Rural	186	44.4
**Marital status**		
Single	35	8.4
Married	251	59.9
Widowed	33	7.8
Divorce	100	23.9
**Ethnicity**		
Amahra	304	72.6
Oromo	16	3.8
Shinasha	15	3.6
Gumuz	10	2.4
Kembata	14	3.3
Agew	56	13.4
Tigre	4	1.0
**Monthly income**		
<1000 birr	143	34.1
1100–3000 birr	102	24.3
3100–4999 birr	76	18.1
>5000 birr	98	23.4
**Occupation**		
Unemployed	30	7.2
Governmental employer	62	14.8
House wife	111	26.5
Daily laborer	88	21
Merchant	55	13.1
Farmer	73	17.4
**Level of education**		
Unable to read & write	111	26.5
Able to read & write	181	43.2
Primary &secondary	93	22.2
College & university	34	8.1

### Knowledge of condom use and drug resistance virus among study participants

Nearly all (98.6%) of study participant were heard /had information about condom. More than half (53%) of respondents had information about drug resistance virus. Among study participants, 228(54.4%) had information about re–infection by drug resistance virus. Of this, 149 (65.1%) respondents were believe that use of condom was the solution for re -infection by drug resistance virus followed by 80(34.9%) were believe that abstinence was the better solution to prevent re-infection (Table 2).

**Table 2:** Knowledge of condom use and drug resistance virus among ART clients in Pawi general hospital, North West Ethiopia 2020 (n = 419).

Variable	Frequency	Present
**Heard about condom**		
Yes	413	98.6
No	6	1.4
**Is there drug resistant virus in the community?**		
Yes	222	53.0
No	197	47.0
**Solution for re-infected and drug resistant virus**		
Abstinence	80	34.9
Condom use	149	65.1

### Sexual behavior and condom use among participants

Most of the respondents were sexually active. Nearly all (90.7%) of the respondents had had sex after they knew sero-status positive, of which 248(65.3%) had sex with their regular spouse/cohabit partner and half of (50.3%) respondents had used condom during sexual intercourse in the last six months.

Nearly three-forth (70.5%) of sexually active respondents had sex with only one sexual partner in the last six months. Among those condom users, nearly half (49.7%) of respondents had never used condom at all. About two-third (64%) of participants were strongly agreed that all HIV positives people should have to use condom during sexual intercourse.

The main reasons for inconsistent use and non-use of condom at all were their assumption that they had the same type of virus 59(31.2%) followed by already infected 44(23.3%), reducing sexual satisfaction 19(10%), desire to have children 34(18%), religious restriction 14(7.5%) and no condom access 19(10%). Three-forth (75.5%) of sexually active respondents have had discussion before sexual intercourse about her HIV sero-status and safe sex. More than half (52.5%) of respondents were drink alcohol and 216(51.6%) were get condom at health facility (Table 3).

**Table 3:** Sexual behavior and condom use among ART client in Pawi general hospital, North West Ethiopia, 2020 (n = 419).

Variable	Frequency	Present
**Had you made sex after you know sero-status positive**		
Yes	380	90.7
No	39	9.3
**Number of partners within the last 6 month**		
None	64	16.8
1	268	70.5
≥2	48	12.6
**Type of partners**		
Steady	248	65.3
Causal	113	29.7
Commercial sex worker	19	5
**Did you discuss about your HIV status before sexual intercourse**		
Yes	282	74.2
No	98	25.7
**Do you think that HIV positive married couples or all HIV positives have to be use condom?**		
Yes	268	64
No	151	36
**Did you use condom in your last sexual intercourse?**		
Yes	191	50.3
No	228	49.7
**Reason for not using condom**		
My wife and me are already infect	44	23.3
We have the same type of virus	59	31.2
Desire to have child	34	18
It decreases sexual satisfaction	19	10
No condom access	19	10
My religion condemn it	14	7.5

### Proportion of consistent condom use

**Fig 1:**
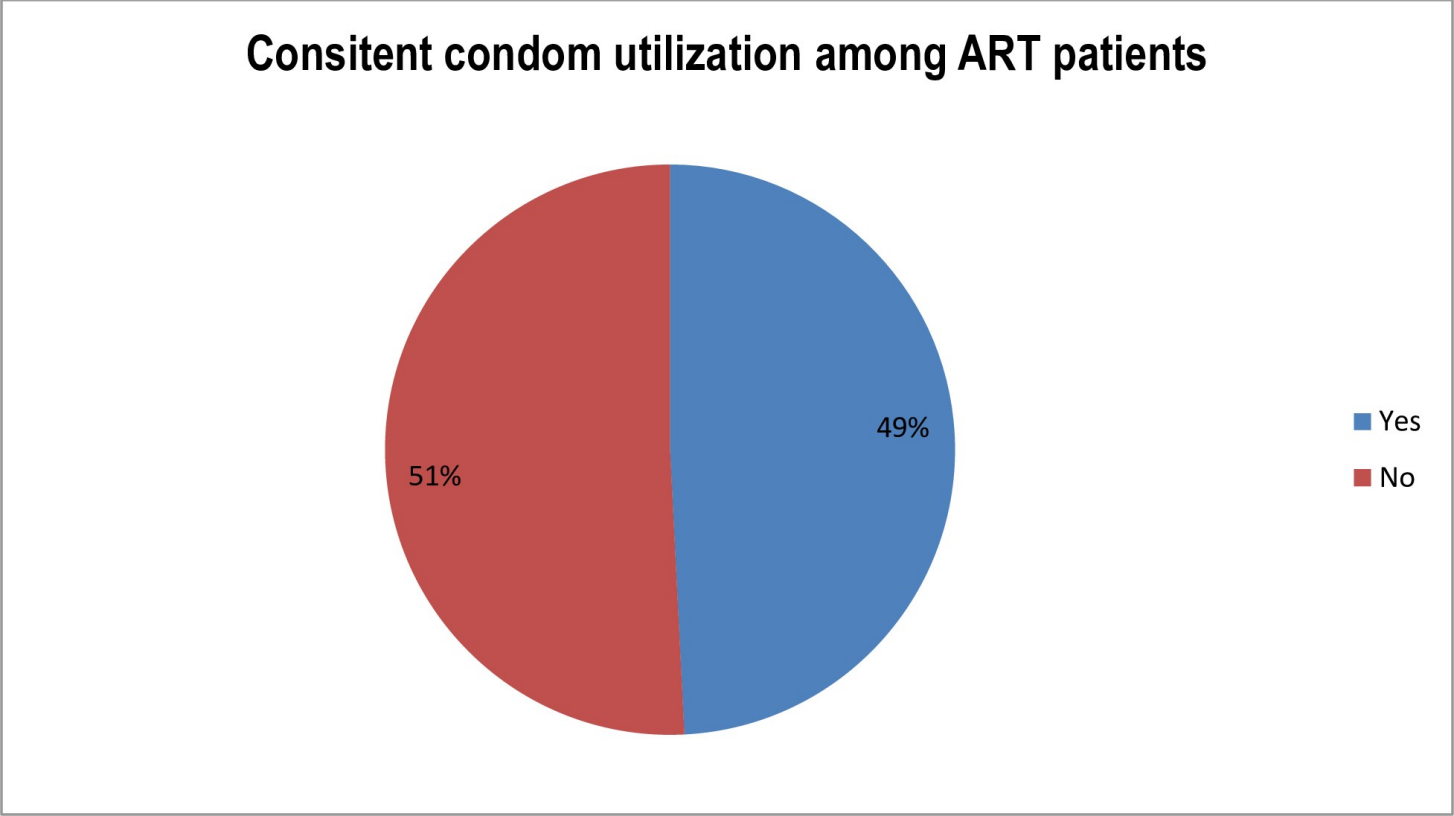
Consistent condom utilization among ART clients in pawi general hospital, North West Ethiopia, 2020 (n = 419).

The study finding showed that among sexually active respondents, 49.2% (95% CI: 42.2–56.5%) had used condom consistently (Fig 1). Among consistent condom users, 59(48%) were females, 46(62.2%) were age range from 25–34, 73 (57%) were urban and 61(66.3%) were able to read and write. The main reason for always condom use was to prevent transmission of other infection like STIs 51(54.3%), followed by to prevent pregnancy (27.7%) and to prevent acquiring and transmitting drug resistant HIV infection (18%).

### Factors affecting consistence condom utilization

In Binary analysis, age, place of residence, level of education and, marital status, number of partner, discuss before sexual intercourse about condom, was associated with consistent condom use. However place of residence, marital status, number of partner and level of education has been found to significantly association in multi -variable analysis with consistent condom use. Respondents in urban residence were **2.16** times more likely to use condom consistently than respondents in rural residence (AOR = 2.16, 95% CI: 1.05, 4.45). The chance of consistent condom use by married was **0.19** times less likely than their counterparts (AOR = 0.19, 95%CI: 0.05, 0.67).

The odds of Consistent condom use among level of informal education were **5.33** times more likely than those educated at college and university (AOR = 5.33, 95% CI: 1.57, 18.08). The likely hood of consistent condom use among those who have one partner were **0.2** times less likely than their counter parts (AOR = 0.2, 95% CI: 0.07, 0.55) (Table 4).

**Table 4:** Binary and multivariable logistic regression analysis with consistent condom utilization among HIV infected persons on ART clinic at Pawi general hospital, North West Ethiopia 2020 (n = 419).

Variable	Consistent condom use		COR 95%	AOR 95%	p-value
	Yes	No			
Age					
18–24	23(65.7%)	12(34.3%)	1	1	
25–34	36(56.3%)	28(43.7%)	0.671(0.285,1.577)	1.028(0.366,2.889)	
35–44	20(39.6%)	31(60.4%)	0.337(0.137,0.825)	0.613(0.214,1.752)	
≥45	15(36.6%)	26(63.4%)	0.301(0.117,0.774)	0.450(0.146,1.385)	
**Residence**					
Urban	73(57%)	55(43%)	2.655 (1.414,4.984)	2.163(1.053,4.447)	0.036
Rural	21(33.3%)	42(66.7%)	1	1	
**Marital status**					
Single	23(81.1%)	5(17.9%)	1	1	
Married	40(36.4%)	70(63.6%)	0.124(0.044,0.352)	0.192(0.055,0.666)	0.009
Widowed	7(50%)	7(50%)	0.217(0.217,0.905)	0.265(0.048,1.455)	
Divorced	24(61.5%)	15(38.5%)	0.348(0.109,1.112)	0.378(0.100,1.426)	
**Level of education**					
Unable to read & write	13(29.5%)	31(70.5%)	0.839 (0.259, 2.715	1.165(0.306,4.434)	
able to read & write	61(66.3%)	31(33.7%)	3.935 (1.35, 11.49)	5.328(1.570,18.081)	0.007
Primary &secondary	14(37.8%)	23(62.2%)	1.217 (0.037, 3.978)	1.451(0.370,5.696)	
College &university	6(33.3%)	12(66.7%)	1	1	
**Discuss before sex about condom**					
Yes	32(65.3%)	17(34.7%)	1	1	
No	62(43.7%)	80(56.3%)	2.429(1.236,4.772)	1.364(0.523,3.557)	
**Number of partner**					
None	30(78.9%)	8(21.1%)	1	1	
1	44(36.4%)	77(63.6%)	0.152(0.064,0.361)	0.197(0.070,0.551)	0.002
≥2	20(49.2%)	12(37.5%)	0444(0.154,1.281)	0.318(0.091,1.116)	

## Discussion

This study showed that consistent condom utilization among ART clients in Pawi general hospital was 49.2% (95% CI: 42.2–56.5), which in line with previous studies conducted in Nigeria 52% [4], South Africa 56% [6], Mekelle Ethiopia (45%) [3] and Koladiba (55.8%) [11]. However, this finding is lower when compared to previous studies conducted in Europe [59%], Kenya [57.4%], Cambodia [80%], Gondar [78.9%] and Jimma [62%] [1, 7, 18–20]. This difference might be due to low educational status of the rural population in the study area and also might be due to lack of professional’s commitment to build awareness on PLWHA on ART to use condom consistently. Furthermore, this study is higher than study conducted in Cameron 29% [21]. This could be explained by different variation in sources of information, characteristics of study participants and the study area.

Almost half of study participants (49.7%) were never use condom at all in the last sexual intercourse. This finding in line with previous study conducted in south eastern Nigeria but lower than study conducted Mekelle [3] and koladiba [11]. This contradiction might be due to the difference in the study area where this study was incorporated study participants from urban and rural area. Furthermore, majority of study participant’s educational level was unable to read and write (26.5%) and access to get information through media, provision of counseling during follow up and knowledge toward use of condoms may be lower.

In this study, 48% of female HIV positive client was used condom consistently. This finding was lower when compared with previous studies conducted in Cameroon (77.5%) [15], Gondar (69%) [1] and koladiba (76%) [11]. This could be due to lack of women decision making power for the use of condom during sexual intercourse. This also might reflect a male resistance to use condoms and lack of awareness to the importance of condom for HIV positive individual. Previous study reported that women’s are feel embarrassed to ask their partners to use condoms and their partner never using condom because they assume that it decrease sexual satisfaction and they went to have child [21].

Important variables significantly associated with consistence condom use in multi-variable in the study were place of residence, marital status, level of has been found to association with consistent condom use. Consistent use of condom was significantly higher among urban residents as compared to their counterparts, which is consistent with previous studies [3, 22–24]. This disparity might be due to that health institutions may increase general awareness about the importance of consistent condom use which target in high risk people who live in and around the urban.

The chance of consistent condom use by married respondent was less likely when compared to single. This finding is supported by the study conducted in Nigeria [24] and Democratic Republic of Congo [25], where condoms were used in all sexual happenstances by unmarried/ single compared to married couples. The possible explanation could be married couples are assumed that no need of condom use during sexual intercourse since we are already infected and may want to have a child.

Educational level of study participant was strongly associated with consistent condom use. The odd of consistent condom use among the study participants who are able to read and write was higher as compared to their counter parts. This finding is similar with previous studies which reported that as educational level advance, the use of condom consistently also increases [24, 26–28]. Moreover, previous finding in sub-Saharan Africa also reported education as a major determining factor of condom use [29].

The likelihood of consistent condom use among those who have one partner was less likely as compared to their counterparts. This study is consistent with previous studies done in South Africa [29] and Democratic Republic of Congo [27]. According to 2016 EDHS report, less than 1% of reproductive age women reported having more than one sexual partner and couples are most often in stable monogamous relationships where condoms are infrequently used [2].

## Conclusion

Consistent condom utilization among HIV positive clients attending ART clinic at Pawi general hospitals was low. The place of residence, marital status, level of education and number of partners were associated factors of consistent condom use. Health education program and counseling services targeted the rural residence and married couples should be started to increase knowledge about way of transmission, merits of consistent condom use and increase self-efficacy towards condom use. Social desirability bias or sensitive nature of question may have led study participant to over-report or under report condom use or other variables. This study also limited to identify on any causal relationship between factors and consistent condom use due to the nature of study design. Therefore, longitudinal studies are recommended to further evaluate this dynamic relationship between the factors and outcomes.

## Supporting information

S1 File Minimal data set of consistent condom use. (SAV)

S2 File English version questionnaire. (PDF)

S3 File Amharic version questionnaire. (PDF)

## Abbreviations

AIDS: Acquired Immune Deficiency Syndrome
AOR: Adjusted Odd Ratio
ART: Anti-Retro Viral Therapy
ARV: Anti-Retro Viral
CI: Confidence Interval
HIV: Human Immune Virus
PLWHA: Peoples living with HIV/ AIDS
SPSS: Statistical Package for Social Science
WHO: World Health Organization

## References

[1] Shewamene Z, Legesse B, Tsega B, Bhagavathula AS, Endale A. Consistent condom use in HIV/AIDS patients receiving antiretroviral therapy in northwestern Ethiopia: implication to reduce transmission and multiple infections. HIV/AIDS (Auckland, NZ). 2015;7:119. doi: 10.2147/HIV.S79122 25926757 PMC4403739

[2] Ababa A. Ethiopia. Abstract available from: https://wfpha.confex.com/wfpha/2012/webprogram/Paper10587.html. 2013.

[3] Yalew E, Zegeye DT, Meseret S. Patterns of condom use and associated factors among adult HIV positive clients in North Western Ethiopia: a comparative cross sectional study. BMC public health. 2012;12(1):308. doi: 10.1186/1471-2458-12-308 22537280 PMC3426486

[4] Akinyemi JO, Awolude OA, Adewole IF, Kanki PJ. Condom use among antiretroviral therapy patients in Ibadan, Nigeria. The Journal of Infection in Developing Countries. 2010;4(08):495–502. doi: 10.3855/jidc.732 20818101

[5] Cicconi P, Monforte AdA, Castagna A, Quirino T, Alessandrini A, Gargiulo M, et al. Inconsistent condom use among HIV‐positive women in the “Treatment as Prevention Era”: data from the Italian DIDI study. Journal of the International AIDS Society. 2013;16(1):18591. doi: 10.7448/IAS.16.1.18591 24135086 PMC3798584

[6] Macharia AG, Kombe Y, Mwaniki P. Consistent condom use among HIV positive women attending comprehensive care centre of thika level 5 hospital, Kenya.

[7] Madiba S, Letsoalo B. Disclosure, multiple sex partners, and consistent condom use among HIV positive adults on antiretroviral therapy in Johannesburg, South Africa. World Journal of AIDS. 2014;4(01):62.

[8] Bennett DE, Bertagnolio S, Sutherland D, Gilks CF. The World Health Organization’s global strategy for prevention and assessment of HIV drug resistance. Antiviral therapy. 2008;13:1. 18578063

[9] EDHS E. demographic and health survey 2016: key indicators report. The DHS Program ICF. 2016.

[10] Tadesse WB, Gelagay AA. Risky sexual practice and associated factors among HIV positive adults visiting ART clinics in public hospitals in Addis Ababa city, Ethiopia: a cross sectional study. BMC Public Health. 2019 Dec;19(1):1–8. doi: 10.1186/s12889-018-6343-3 30691435 PMC6348678

[11] Centers for Disease Control and Prevention. (2014). Condoms and STDs: Fact Sheet for Public Health Personnel. Retrieved from E. www.cdc.gov/condomeffectiveness/docs/

[12] Hughes J. P., Baeten J. M., Lingappa J. R., Magaret A. S., Wald A., de Bruyn G. … Partners in Prevention HSV/ HIV Transmission Study Team. (2012). Determinants of per-coital-act HIV-1 infectivity among African HIV-1-serodiscordant couples. Journal of Infectious Diseases, 205, 358–365. doi: 10.1093/infdis/jir747 22241800 PMC3256946

[13] Grasso M. A., Schwarcz S., Galbraith J. S., Musyoki H., Kambona C., Kellogg T. A.; et al. (2016). Estimating the prevalence and predictors of incorrect condom use among sexually active adults in Kenya: Results from a nationally representative survey. Sexually Transmitted Diseases, 43, 87–93. doi: 10.1097/OLQ.0000000000000393 26766524 PMC4930356

[14] Giannou F. K., Tsiara C. G., Nikolopoulos G. K., Talias M., Benetou V., Kantzanou M., et al. (2015). Condom effectiveness in reducing heterosexual HIV transmission: A systematic review and meta-analysis of studies on HIV serodiscordant couples. Expert Review of Pharmacoeconomics & Outcomes Research, 2016; 16(4), 489–99. doi: 10.1586/14737167.2016.1102635 26488070

[15] Ali MS, Tesfaye Tegegne E, Kassa Tesemma M, Tesfaye Tegegne K. Consistent condom use and associated factors among HIV-positive clients on antiretroviral therapy in North West Ethiopian Health Center, 2016 GC. AIDS research and treatment. 2019 Mar 17;2019. 10.1155/2019/7134908 PMC6441518 31007955

[16] EpiData Association. Epidata Software 3.1.

[17] Mertler CA, Reinhart RV. Advanced and multivariate statistical methods: Practical application and interpretation. Routledge; 2016 Oct 24.

[18] De Cock KM, De Lay P. HIV/AIDS estimates and the quest for universal access. The Lancet. 2008;371(9630):2068–70. doi: 10.1016/S0140-6736(08)60732-1 18571714

[19] Sinding SW. Does’ CNN’(condoms, needles and negotiation) work better than’ABC’(abstinence, being faithful and condom use) in attacking the AIDS epidemic? International Family Planning Perspectives. 2005;31(1):38–40. doi: 10.1363/3103805 15888408

[20] Tuot S, Pal K, Thin K, Patio C, Allbritton K, Blondek C, et al. Determinants of inconsistent condom use among HIV serodiscordant couples in Cambodia.

[21] Tarkang EE. Factors associated with consistent condom use among senior secondary school female learners in Mbonge subdivision of rural Cameroon. Journal of AIDS and HIV Research. 2012 Jun 30;5(6):214–23.

[22] Denue BA, Kwayabura SA, Bukbuk D, Inuwa U, Ajayi BB. Evaluation of Condom Use and Associated Factors among Adult HIV Clients in Maiduguri, North Eastern Nigeria: A Comparative Cross Sectional Study. World Journal of AIDS. 2014;4(02):169.

[23] Mansergh G, Naorat S, Jommaroeng R, Jenkins RA, Stall R, Jeeyapant S, et al. Inconsistent condom use with steady and casual partners and associated factors among sexually-active men who have sex with men in Bangkok, Thailand. AIDS and Behavior. 2006;10(6):743–51. doi: 10.1007/s10461-006-9108-4 16715348

[24] Sunmola AM. Factors associated with consistent condom use by employees in the brewery industry in Nigeria. SAHARA-J: Journal of Social Aspects of HIV/AIDS. 2004 Jul 14;1(1):27–34. doi: 10.1080/17290376.2004.9724824 17600997

[25] Carlos Silvia, Lopez-del Burgo Cristina, Burgueño Eduardo, Martinez-Gonzalez Miguel Angel, Alfonso Osorio, Ndarabu Adolphe, et al.(2016): Male condom use, multiple sexual partners and HIV: a prospective case-control study in Kinshasa (DRC), AIDS Care, doi: 10.1080/09540121.2016.1258450 27852108

[26] Delva W, Meng F, Beauclair R, Deprez N, Temmerman M, Welte A, et al. Coital frequency and condom use in monogamous and concurrent sexual relationships in Cape Town, South Africa. Journal of the International AIDS Society. 2013 Jan;16(1):18034. doi: 10.7448/IAS.16.1.18034 23618365 PMC3636421

[27] Dessie Y., Gerbaba M., Bedru A. and Davey G. (2011) Risky Sexual Practices and Related Factors among ART Attendees in Addis Ababa Public Hospitals, Ethiopia: A Cross-Sectional Study. BMC Public Health, 11, 422. doi: 10.1186/1471-2458-11-422 21631935 PMC3138456

[28] Ncube N.M., Akunna J., Babatunde F., Nyarko A., Yatich N.J., Ellis W., et al. (2012) Sexual Risk Behaviour among HIV-Positive Persons in Kumasi, Ghana. Ghana Medical Journal, 46, 27–33. 22605886 PMC3353501

[29] Agha S. (1998). Sexual activity and condom use in Lusaka, Zambia. International Family Planning Perspectives, 24, 32–37.

