# Supplementary material for: Consistent condom utilization and associated factors among HIV positive clients attending ART clinic at Pawi general hospital, North West Ethiopia
Source: PLoS One. 2021 Dec 21;16(12):e0261581. doi: 10.1371/journal.pone.0261581 (PMC8691638; doi:10.1371/journal.pone.0261581)
Supplement: S2 File — (PDF) [file pone.0261581.s002.pdf]

# English version questionnaire

## I. Information sheet and consent form

**Title of the Research proposal:** consistent condom utilization and associated factors among HIV/AIDS patients in pawi general hospital on ART clinic. Pawi, North West Ethiopia, 2020

Name of Principal Investigator:-Adugna Lencha et al

**Name of the Organization**:-Pawi General Hospital

Introduction

This information sheet and consent form is prepared for HIV positive peoples who have follow up in pawl general hospital during the study period who was participate in the research project. This information sheet and consent form is prepared with the aim of explaining the research project that you are asked to join by the group of research investigators. The main aim of the research project is to determine consistence condom utilization and associated factors among HIV positive peoples who have follow up in pawi general hospital.

**Purpose of the Research Project:** consistence utilization of condom and associated factors among HIV positive peoples who have follow up in pawl district hospital The purpose of this study is to fill the gap and to show the magnitude of condom utilization in HIV positive individuals in pawl general hospital and this was provide valuable information to health care planners to design evidence based policy. The scarcity of research in this area, the emergence of the problem and need of the information by governmental and nongovernmental organizations makes it feasible for the study.

**Procedure** In order to determine consistence condom utilization and associated factors among HIV positive peoples who have follow up in pawi general hospital we invite you take part in our project. If you are willing to participate in the project, you need to understand the purpose of the study and give verbal consent. Then; you was requested to give your response to the data collectors.

**Risk and /or Discomfort** By participating in this research project you may feel some discomfort especially on scarifying your time (about 20-25 minutes) otherwise, no risk in participating in this research project and this may not be too much as you are one of the patient construction sector in the site, so your response provide an important input to show the gap and means to improve the health service and the community.

**Benefits:** If you are participating in this research project, the output of the study was have both direct and indirect benefit to you, but your participation is likely to help us in showing the problems faced by HIV positive individuals and to recommend appropriate intervention.

**Incentives/Payments for Participating:** You were not been provided any incentives or payment to take part in this project.

**Confidentiality:** The information collected from this research project was kept confidential and information about you that was collected by this study was stored in a file, without your name, but a code number assigned to it. And it was not be revealed to anyone except the principal investigator and was kept locked with key.

**Right to Refuse or Withdraw** You have the full right to refuse from participating in this research. You can choose not to respond some or all the questions and this was not affect you from getting any kind of service given to patients in the hospital. You have also the full right to withdraw from this study at any time you wish, without losing any of your right.

## II. **Questionnaire**

This questionnaire was prepared to determine consistence condom utilization and associated factors among patients in antiretroviral follow up clinic pawi general hospital.

Consent form For Participation

Questionnaire number -------------------------------------------------------

Personal -A- Interviewer------------------------------------------------------- Code -------------

- B - Supervision ---------------------------------------------- Code -------------

Data Collection ----------------DD------------MM---------------------YYY-----------------------

Time at the beginning of the interview -----------------------------------------------------------

Time at the end of the interview -----------------------------------------------------------------

My name is -------------------------------------------Working for **Adugna Lencha** et al as a data collector. I conducting a research on the problem of condom utilization and associated factors among patients in antiretroviral follow up clinic pawi general hospital we would appreciate your participation in the study. I would like to ask you about case related questions. The questionnaire was take 20 to 25 minutes to complete. Participation in the interview is entirely on voluntary basis and you can choose not to answer any individual question or all questions. Whatever information you provide was kept strictly confidential was not be shown to other person. Your participation in the study was help to measure the problem and to plan appropriate intervention and it is entirely on voluntary basis and you can choose not to answer any individual question or all questions. However we hope you will participate fully in this assessment since your views are important.

Do you have any questions about the research?

May I begin the Interview now? 1. Yes 2. No

INTERVIEW QUESTIONER ON CONSISTENCE CONDOM UTILIZATION AND ASSOCIATED FACTORS AMONG HIV POSITIVE INDIVIDUALS ON FOLLOW UP CARE IN PAWI GENERAL HOSPITALE ARV TREATMENT UNITS.

Section I: Socio economic demographic characteristics

| No | Questions | Response |
|---|---|---|
| *101* | How old are you? | __________ years (age in completed years) |
| 102 | Sex | 1.male<br>2.female |

| | | |
|---|---|---|
| 103 | What is your education level? | 1. un able to read and write<br>2. able to read and write<br>3. primary<br>4. high school<br>5. College or university |
| 104 | What is your ethnicity? | 1. Amhara<br>2. .shinasha<br>3. Oromo<br>4. Gumuz<br>5. other specify ____________ |
| 105 | What is your religion? | 1. Orthodox.<br>2. . Muslim.<br>3. Protestant.<br>4. Catholic<br>5. other specify ______________ |
| 106 | What is your current Marital status? | 1. Single.<br>2. Married<br>3. Divorce<br>4. Separated<br>5. Widowed |
| 107 | What is your Occupation? | 1.unemployed<br>2.employed<br>3. house wife<br>4.farmer<br>5. daily laborer<br>6. merchant<br>7. military<br>8. other specify______________ |
| 108 | How much is your total monthly income? | Specify____________________ |

**Section II** : **Information on condom and knowledge of drug resistance virus**

| | | | |
|---|---|---|---|
| 201 | Have you heard about condom? | 1. Yes<br>2. .No | |
| 202 | Do you think that there is drug resistant virus in the community? | 1. Yes<br>2. No | |
| 203 | Do you think that HIV positive peoples will be re –infected by a resistant virus? | 1. Yes<br>2. No | If No skip to 205 |
| 204 | If your answer is yes, what is the solution? | 1.Abstinence<br>2.Condom use<br>3. Other___________ | |

**Section III – Sexual behavior related questions**

| | | | |
|---|---|---|---|
| 301 | Have you ever had sex after you have heard that you are HIV positive? | 1. Yes<br>2. No | |
| 302 | How many partners did you have within the past 6 month? | Specify --------- | |
| 303 | What type of partner did you have? | 1. Stable<br>2. Commercial sex worker<br>3. Casual<br>4. I have all type | |
| 304 | Have you discus before about your HIV status with your sexual partner | 1. Yes<br>2. No | |
| 305 | Do you think that HIV positive married couples or all HIV positives have to be use condom? | 1. Yes<br>2. No | If yes skip to 307 |
| 306 | If your answer is No, would you tell me the reason please? | 1. My wife and me are already infected.<br>2. We have the same type of virus.<br>3. We want to have a child.<br>4. It decreases my sexual satisfaction.<br>5. There is no condom access<br>6. My religion does not permit it<br>Others ----------------- | |

| | | |
|---|---|---|
| 307 | Have you used condom in your last sexual intercourse? | 1. yes<br>2. No |
| 308 | Do you always use condom during sexual intercourse?. | 1. yes<br>2. No |
| 309 | If your answer is yes why you are using. | 1. To prevent others from infection.<br>2. For family planning.<br>3. To prevent acquiring and transmitting drug resistant HIV<br>4. other specify ________ |
| 310 | Do you use the following | Alcohol<br>khat<br>shisha<br>other specify |
| 311 | Where do you think one can get condom better place if he/she wants to use it. | 1. Health facility<br>2. Pharmacy<br>3. Shop<br>4. from HIV positive associations |

**Thank you for your devotion to complete this questioner**

Interviewer:___________________Signature:_______ Date:_________ Time:________
