# Supplementary material for: Consistent condom utilization and associated factors among HIV positive clients attending ART clinic at Pawi general hospital, North West Ethiopia
Source: PLoS One. 2021 Dec 21;16(12):e0261581. doi: 10.1371/journal.pone.0261581 (PMC8691638; doi:10.1371/journal.pone.0261581)
Supplement: S3 File — (PDF) [file pone.0261581.s003.pdf]

አማርኛ የተዘጋጀ መጠይቅ

ፀረ ኤችአይ ቪ መድሀኒት ክትትል ለሚያደርጉ ታካሚዎች የተዘጋጀ መጠየቅ

ጤና ይስጥልኝ። ስሜ ………………………….ይባላል። ይህ ጥናት በባህር ዳር ዩኒቨርሲቲ የማስተርስ ዲግሪ በክሊኒካል ሚድዋፍሪ የትምህርት ዘርፍ ተማሪ በሆነው አዱኛ ሌንጫ የሚደረግ ነዉ። ይህ መጠይቅ የተዘጋጀው በፓዌ ሆስፒታል ፀረ-ኤች አይቪ መድሃኒት ክትትል ክፍል ለሚከታተሉ በሽተኞች ኮንዶምን በምን ያህል መጠን መጠቀማቸውን እና ተያያዥ ችግሮችን ለማወቅ *ነው።* የጥናቱ ተሳታፊዎች የሚመረጡት በዕጣ ነው። ስለዚህ እርስዎም የተመረጡት በዕጣ ነው። እርስዎ የሚሰጡን መረጃዎች ለአገልግሎት አቅርቦትና ጥራት መሻሻል ከፍተኛ አስተዋጽኦ ያበረክታሉ። በጥናቱ ወቅት እርስዎ የሚሰጡን ማናቸውም አይነት መረጃዎችለዚህ ጥናት ብቻ ይውላሉ ለሌላ አካል ተላልፈው አይሰጡም። የእርስዎ ስም ወይም ማንነት አይመዘገብም በምትኩ መለያ ቁጥር የሚሰጠው ይሆናል። ማንኛውም በዚህ ጥናት የሚሳተፍ ወይም በዚህ ጤና ድርጅት ውስጥ የሚሰራ ሰው ስለእርስዎ ምንም አይነት መረጃ አይነገረውም።በጥናቱ ላይ ያለመሳተፍ፣ በፈለጉት ሰዓት መጠይቁን የማቋረጥና መመለስ የማይፈልጉትን ጥያቄዎች የመተው ወይም ያለመመለስ ሙሉ መብት አለዎት። በጥያቄው መሳተፍዎም ሆነ ያለመሳተፍዎ በሚያገኙት የጤና አገልግሎት ላይ የሚኖረው አዎንታዊም ሆነ አሉታዊ ተፅዕኖ የለም። ቃለ መጠይቁን በሙሉ ለማጠናቀቅ በአማካይ 20-30 ደቂቃ ያህል ይፈጃል። ከእርስዎ የሚገኘው መረጃ መንግስትና ሌሎች ድርጅቶች የአቅርቦቱን ጥራት ለማሻሻል ለሚያደርጉት እንቅስቃሴ ከፍተኛ አስተዋጽኦ ና ድጋፍ ይኖረዋል:; ተጨማሪ መረጃ ከፈለጉ ከታች ባለው አድራሻ መጠየቅ ይችላሉ።

አዱኛ ሌንጫ +251928258451/0905289794 adugnaadugna09@gmail.com

ለመሳተፍ ፍቃደኛ ነዎት? አዎን ☐ አይደለሁም ☐

የቃለ መጠየቅ መለያ ቁጥር ……………………

የጥናቱ ቦታ ፡ ፓዌ አጠቃላይ ሆስፒታል

የጠያቂው ስም -------------------- መለያ ቁጥር---------------------

የተቆጣጣሪው ስም ------------------------------.

መጠየቅ የተደረገበት ቀን ------------ ወር -------- ዓ/ም-------------

መጠየቁ የተጀመረበት ስዓት ------------. ያለቀበት ስዓት --------------

የሚከተሉት ጥያቄዎች ምርጫ ያላቸው እና መልስ ነው ያሉትን ያክብቡ ለሌላቸው ክፍት ቦታውን ይሙሉ

ተ/ቁ	**መጠይቅ**	**ምላሽ**	
**101**	ዕድሜ	---------- (በሙሉ አመት ይገለፅ)	
**102**	ጾታ	**1.** ወንድ<br>**2.** ሴት	
**103**	የትምህርት ደረጃ	**1.** ማንበብና መፃፍ የማይችል<br>**2.** ማንበብና መፃፍ የሚችል<br>**3.** **1**ኛ ደረጃ ትምህርቱን ያጠናቀቀ<br>**4.** የ**2**ኛ ደረጃ ትምህርት ያጠናቀቀ<br>**5.** የኮሌጅ ወይንም የዩኒቨርስቲ ት/ቱን ያጠናቀ/ች	
**104**	ብሄረሰብ	**1.** አማራ<br>**2.** ሽናሻ<br>**3.** ኦሮሞ<br>**4.** ጉምዝ<br>**5.** ሌላ ካለ ይጠቀስ-------------.	
**105**	ሃይማኖት	**1.** ኦርቶዶክስ<br>**2.** እስልምና<br>**3.** ፕሮቴስታንት<br>**4.** ካቶሊክ<br>**5.** ሌላ ካለ ይጠቀስ	
**106**	የጋብቻ ሆኔታ	**1.** ያገባ/ች<br>**2.** የፈታ/ች<br>**3.** ያገባ/ች<br>**4.** የሞተበት/ት	
**107**	ስራ	**1.** ስራ የሌለው<br>**2.** ስራ ላይ ያለ<br>**3.** የቤት እመቤት<br>**4.** አርሶ አደ**C**	

		5. የቀን ሰራተኛ<br>6. ነጋዴ<br>7. ወታደር<br>8.ሌላ ካለይጠቀስ ------------------	
**108**	የወር ገቢ	መጠኑ ይገለ---------------------	
ክፍል ሁለት ኤች አይቪን የሚመለከቱ ጥያቄዎች<br>***የሚከተሉት ጥያቄዎች ምርጫ ያላቸው እና መልስ ነው ያሉትን ያክብቡ***			
**201**	ስለ ኮንዶም ሰምተው ያውቃሉ	1. አዎ<br>2. የለም	
**202**	ፀረ-ኤች አይቪ መድሃኒት የተለማመደ ቫይረስ በማህበረሰባችን ውስጥ አለ ብለው ያምናሉ	1. አዎ<br>2. የለም	
**203**	ኤች አይቪ ቫይረስ በደማቸው ውስጥ ያለ ሰዎች በፀረ-ኤች አይቪ የተለመደ ቫይረስ ደግሞ ይያዛሉ ብለው ያምናሉ	3. አዎ<br>1. የለም	የለም ከሆነ ወደ ቁጥር 205
**204**	መልሰዎ አዎ ከሆነ መፍትሄው ምንድ ነው ይላሉ	1. መታቀብ<br>2. ኮንዶም መጠቀም<br>3. ሌላ ካለ ይጠቀስ----------	
ክፍል ሶስት<br>***ወሲባዊ ባህሪያትን የተመለከተ ጥያቄዎች የሚከተሉት ጥያቄዎች ምርጫ ካላቸውን ትክክለኛውን መልስ ያለ ካለ ትክክለኛውን መልስ ያክብቡ ምርጫ የሌላቸውን ክፍት ቦታ ይሙሉ***			
**301**	ኤች አይቪ በደሞ ውስጥ መኖሩን ካወቁ በኃላ የግብረ ስጋ ግንኙነት አርገው ያዉቃሉ	1. አዎ<br>2. የለም	
**302**	ባለፉት **6** ወራት ስንት የወሲብ አጋር ነበረዎት	-------------------------------------	
**303**	የዕርሰዎ የወሲብ አጋር ከሚከተሉት ውስጥ የትኛው ነዉ	1. ቋሚ የወሲብ ጓደኛ<br>2. ከሴተኛ አዳሪ<br>3. ድንገተኛ የወሲብ ጓደኛ<br>4. ሌላ ካለ..........	
**304**	ከአሁን በፊት ስለኤች አይቪ ውጤተዎት ከወሲብ አጋረዎ ጋር ተወያይተው ያውቃሉ	1. አዎ<br>2. የለም	

**305**	ትዳር ላይ ሆነው ኤች አይቪ ቫይረስ ያላቸው ሰዎች ወይም የወሲብ ጓደኛሞች ኮንዶም መጠቀም አለባቸው ብለው ያምናሉ	**1.** አዎ<br>**2.** የለም	
**306**	መልሰዎ የለም ከሆነ ምክንያቱ ምንድነው ?	**1.**እኔና ባለቤቱ ቫይረሱ ስላለብን አያስፈልገንም<br>**2** **.**አንድ አይነት ቫይረስ ስለያዘነ አያስፈልገንም<br>**3.** ልጅ መውለድ ስለሚያስፈልገን<br>**4.** ኮንዶም የወስብ እርካታየን ስለሚቀንስብኝ<br>**5.** ኮንዶም በቅርብ ስለማላገኝ<br>**6.** ሃይማኖታችን ስለማይፈቅድ	
**307**	አሁን መጨረሻ ላይ የወስቢ ግንኙት ሲፈፅሙ ኮንዶም ተጠቅመዋሌ	**1.** አዎ<br>**2.** የለም	
**308**	ወሲብ በሚፈፅሙበት ሰአት ሁልጊዜ ኮንዶም ይጠቀማሌ	**1.** አዎ<br>**2.** የለም	
**309**	መልሰዎ አዎ ከሆነ ምክንያተዎ ምንድን ነዉ	**1.** ኤች አይቪን ወደ ሌላ ሰው ላለማስተላለፍ<br>**2.** ለወሊድ መቆጣጠሪያ<br>**3.** ፀረ-ኤችአይቪ መድሃኒት የለማመደቫይረስ እንዳይተላለፍብኝ<br>4 ሌላካለ.........	
**310**	የሚከተሉትን ነገሮች ተጠቅመው ያውቃሉ	**1** አልኮል መጠጥን<br>**3.** ጫት<br>**4.** ሽሻ<br>**5.** ሌላ ካለ ይጠቀስ__________	
**311**	ኮንዶምን ከፈለጉ ለማግኙት አመች በታ የትኛው ነው	**1.** ጤና ተቋም<br>**2.** ፋርማሲ<br>**3.** ሱቁ<br>**4.** ቫይረሱ በደማቸው ውስጥ ካለ የህብረተሰብ ክፍል ማህበራት	

		ውስጥ	

ጊዜዎን ሰውተው መልስ ስለሰጡን እጅግ በጣም አድርገን እናመሰግናለን።
